# Preclinical Models for Acquired Resistance to Third-Generation EGFR Inhibitors in NSCLC: Functional Studies and Drug Combinations Used to Overcome Resistance

**DOI:** 10.3389/fonc.2022.853501

**Published:** 2022-04-07

**Authors:** Emna Mahfoudhi, Charles Ricordel, Gwendoline Lecuyer, Cécile Mouric, Hervé Lena, Rémy Pedeux

**Affiliations:** ^1^ Univ Rennes, Institut Nationale de la Santé et de la Recherche Médicale (INSERM), COSS (Chemistry Oncogenesis Stress Signaling), UMR_S 1242, Centre de Lutte Contre le Cancer (CLOC) Eugène Marquis, Rennes, France; ^2^ Centre Hospitalier Universitaire de Rennes, Service de Pneumologie, Université de Rennes 1, Rennes, France

**Keywords:** preclinical models, 3rd G EGFR-TKI, resistance mechanism, osimertinib, lung cancer

## Abstract

Epidermal growth factor receptor (EGFR)-tyrosine kinase inhibitors (TKIs) are currently recommended as first-line treatment for advanced non-small-cell lung cancer (NSCLC) with *EGFR*-activating mutations. Third-generation (3rd G) EGFR-TKIs, including osimertinib, offer an effective treatment option for patients with NSCLC resistant 1st and 2nd EGFR-TKIs. However, the efficacy of 3rd G EGFR-TKIs is limited by acquired resistance that has become a growing clinical challenge. Several clinical and preclinical studies are being carried out to better understand the mechanisms of resistance to 3rd G EGFR-TKIs and have revealed various genetic aberrations associated with molecular heterogeneity of cancer cells. Studies focusing on epigenetic events are limited despite several indications of their involvement in the development of resistance. Preclinical models, established in most cases in a similar manner, have shown different prevalence of resistance mechanisms from clinical samples. Clinically identified mechanisms include EGFR mutations that were not identified in preclinical models. Thus, NRAS genetic alterations were not observed in patients but have been described in cell lines resistant to 3rd G EGFR-TKI. Mainly, resistance to 3rd G EGFR-TKI in preclinical models is related to the activation of alternative signaling pathways through tyrosine kinase receptor (TKR) activation or to histological and phenotypic transformations. Yet, preclinical models have provided some insight into the complex network between dominant drivers and associated events that lead to the emergence of resistance and consequently have identified new therapeutic targets. This review provides an overview of preclinical studies developed to investigate the mechanisms of acquired resistance to 3rd G EGFR-TKIs, including osimertinib and rociletinib, across all lines of therapy. In fact, some of the models described were first generated to be resistant to first- and second-generation EGFR-TKIs and often carried the T790M mutation, while others had never been exposed to TKIs. The review further describes the therapeutic opportunities to overcome resistance, based on preclinical studies.

## Introduction

Epidermal growth factor receptor (*EGFR*)-activating mutations in non-small-cell lung cancer (NSCLC) are an important predictor of treatment efficacy with EGFR tyrosine kinase inhibitors (TKIs). EGFR-TKIs have been shown to prolong the survival of patients with tumors harboring *EGFR*-activating mutations from less than 1 year to approximately 20 to 30 months (1, 2). Despite the initial benefits, almost all tumors develop resistance leading to disease progression. Acquired resistance against the first- and second-generation EGFR-TKIs is primarily caused by the development of the secondary EGFR^T790M^ mutation (3, 4). Several third-generation EGFR-TKIs have been developed to overcome T790M-induced resistance. AZD9291 (osimertinib), CO-1686 (rociletinib), HM61713 (olmutinib), EGF816 (nazartinib), ASP8273 (naquotinib), lazertinib (YH25448), PF06747775, and AC0010 (avitinib) are third-generation (3rd G) EGFR-TKIs, which selectively and irreversibly inhibit EGFR with the common activating mutations, exon 19 deletion (Del19) and exon 21 L858R mutation, and the T790M mutation while sparing wild-type EGFR (5, 6). Currently, osimertinib is the standard of care for EGFR-positive advanced NSCLC with T790M mutation. It has been shown to have remarkably positive results as a first-line treatment for EGFR-mutated advanced NSCLC, with a median progression-free survival (PFS) of 18.9 months (7), leading to its approval for first-line treatment of metastatic EGFR-mutated NSCLC.

Preclinical modeling and analysis of tumor tissue obtained from patients after disease progression have led to the identification of many mechanisms involved in resistance. Contrary to 1st- and 2nd-generation TKIs, no predominant gatekeeper-resistant gene mutations have been observed (8). In clinical studies, mechanisms responsible for resistance include the emergence of mutations in exon 20 of EGFR (e.g., C797S) (9), MET and HER2 amplification, gene fusion, altered cell cycle genes, and *de novo* mutations in KRAS (10).

The pattern of resistance mechanisms differs in the reported clinical and preclinical studies. The vast majority of preclinical models developed to date to identify the mechanisms underlying resistance to 3rd G EGFR-TKIs have used sensitive cell line models exposed to the drug until resistance emerges. The drug concentrations and exposure duration vary from study to study. However, despite the large number of models available, 40% to 50% of the genetic mechanisms associated with disease progression during osimertinib treatment are still unknown (11). This raises the question of whether continuing to generate preclinical models on a recurrent basis would really help to better decipher the mechanisms involved in resistance acquisition and discover biomarkers of relapse. Resistance to EGFR-TKIs therapy is associated with high tumor heterogeneity (12). Such heterogeneity requires tools that mimic the real world for the discovery and evaluation of new therapeutic strategies.

Recent published reviews on resistance mechanisms have focused, in particular, on clinical studies (13–18). Only one review specifically addressed preclinical models, but only those generated from cell lines resistant to first-line osimertinib (19). In this review, we describe reported preclinical models established to identify mechanisms responsible for or involved in resistance to 3rd G EGFR-TKIs and the combinatorial approaches used to circumvent this resistance. We also highlight whether the identified mechanism has been reported in clinical studies. In **Table 1**, the models are listed in the order in which they are cited in the review. Drug doses or the duration of treatment are not listed if not reported in the original articles or references. Models based on modified cell lines generated by transfection, transduction, and/or site-directed mutagenesis are not included in **Table 1**, but are mentioned in the review.

**Table 1:** Preclinical models of resistance to third-generation EGFR-TKIs.

Model generation method	Cell line	3rd G TKI	Genetic alteration	Therapy	Method/approach	References
**On-target EGFR-dependent mechanisms**						
Dose escalation method (0.3 to 1 μM) for several months	PC9	Rocilitinib	EGFR amplification	Cetuximab + rocilitinib Afatinib + rocilitinib	FISH, Exome sequencing, DNA qPCR	Nukaga et al. (20)
**MAPK/PI3K implication**						
- Chronic treatment with osimertinib single dose 160 nM for several months -Dose escalation method until 160 nM osimertinib -Dose escalation method until 1500 nM WZ4002 or osimertinib	PC9 and H1975	Osimertinib WZ4002	- NRAS gain - NRAS Q61K, NRAS E63K and NRAS G12V/R - KRAS gain - MAPK1 gain - CRKL1 gain	Selumetinib (MEK inhibitor) + osimertinib	SnaPshot mutation analysis, targeted and whole exome sequencing	Eberlein et al. (21)
Dose-escalation exposure (0.01 to 1.0 µmol/L) for 7.8 months followed by single-cell cloning	Gefetinib resistant PC9(T790M +)	Naquotinib	NRAS amplification in all sub-clones	Selumetinib/Trametinib (MEK inhibitor) + naquotinib	RNA kinome sequencing, WB, qPCR, and NRAS-GTP pull-down	Ninomiya et al. (22)
Escalation dose steps (0.3 to 1 μM)	PC9	Osimertinib	KRAS G13D	ND	Whole-exome sequencing (WES)	Nukaga et al. (20)
Exposure to increasing concentration (10 nM to 1 μM)	PC9	Osimertinib	HRAS G13R with increased MET expression	ND	NGS	Ku et al. (23)
Exposure to increasing concentration (10 to 500 nM) followed by cloning	PC9	Osimertinib	BRAF G469	Selumetinib/Trametinib + osimertinib	NGS	La Monica et al. (24)
Dose escalation method (0.3 to 1 μM)	H1975	Osimertinib	Integrin β1 and phospho-Src upregulation with EMT	Dasatinib/bosutinib (src inhibitor) + osimertinib	WB and Q-PCR	Nukaga et al. (20)
**MET alterations**						
- PC9 mice xenograft tumors collected after 100 days of rociletinib administration (150 mg/kg BID) - L858R-positive patient-derived xenograft	–	Rocilitinib	MET amplification	Crizotinib + rocilitinib	CAPP-Seq profiling, NGS, RTK array, FISH	Chabon et al. (25)
Exposure to increasing concentrations (10 nM to 500 nM) for approximately 6 months	HCC827	Osimertinib Cross resist to CO-1686 and erlotinib	MET copy gain	ARQ179/ SGX523 / crizotinib (MET inhibitors) + osimertinib	WB, qPCR	Shi et al. (26)
Resistant clones were generated by cloning of Resistant cell populations established from resistant xenograft tumors obtained after a series of continuous drug exposure for 115 days.	H1975	AC0010 Cross- resist to CO-1686 and to osimertinib	MET upregulation	Crizotinib + AC0010	RNA-sequencing, WB	Xu et al. (27)
Exposure to increasing concentrations (0.01 to 1.0 µmol/L) during 5.2 months	PC9	Naquotinib	MET amplification	Crizotinib/SGX523 + naquotinib	Phospho-RTK arrays, WB, FISH	Ninomiya et al. (22)
**AXL**						
Resistant cells were established from subcutaneous tumors collected from mice treated for 29 days with osimertinib	PC9	Osimertinib	AXL overexpression	ONO-7475 (AXL inhibitor) + osimertinib	WB	Okura et al. (28)
						
Stepwise escalation up to 3 μM	H1975	Osimertinib	STC2 upregulation AXL overexpression	R428 (AXL inhibitor) + osimertinib	WB, qPCR, phospho-RTK array	Liu et al. (29)
Exposure to escalating doses (0.001–0.5 μM)	HCC827	Osimertinib	GAS6 overexpression AXL overexpression	YD (degrader) + osimertinib	WB, IHC	Kim et al. (30)
- Exposure to stepwise escalation (10 nmol/L to 2 μmol/L) over 6 months - Exposure intermittently to 2 μmol/L over 6 months	HCC827, HCC4006, PC-9, H1975	Osimertinib	AXL upregulation AXL upregulation+ EMT+ EGFR copy loss+ ALDHA1 upregulation AXL upregulation+ MET amplification	Cabozantinib (TKIs inhibitor including AXL) + osimertinib	WB, NGS, qPCR	Namba et al. (31)
Exposure to increasing doses up to 1 μM for more than 6 months	HCC827	Osimertinib	AXL upregulation associated with MET amplification	CB469 (dual MET and AXL inhibitor) + osimertinib	Phospho-RTK-array	Yang et al. (32)
Stepwise dose escalation	Gefitinib-resistant PC9 (T790M+)	Osimertinib	AXL overexpression AXL overexpression with MET activation FGFR1 upregulation	-Foretinib (RTK and AURKB inhibitor) -Barasertib (AURKB-specific inhibitor) -Tozasertib	WB, IHC and Q-PCR	Bertran-Alamillo et al. (33)
**IGF1-R**						
Exposure to increasing concentrations	Gefitinib-resistant PC9	WZ4002	IGF1-R activation with IGFBP3 decreased expression	AG-1024 (IGF1-R inhibitor) or BI836845 (monoclonal anti-IGF1/2 blocking antibody) + WZ4002	RTK-array	Park et al. (34)
Stepwise escalation method from 150 nmol/L to 1 μmol/L over 6 months - Chronic exposure to 1 μmol/L over 3 months	- Gefitinib-resistant PC9 (T790M+) - H1975	Osimertinib	IGF1R activation	Linsitinib (IGF1R inhibitor) + osimertinib	RTK array	Hayakawa et al. (35)

Dose escalation method (0.03 to 1 μmol/L) for several months followed by cloning	PC9	Osimertinib	IGF1-R activation mediated by IGF2 overexpression	Linsitinib + osimertinib	Phospho-RTK array, ELISA	Manabe et al. (36)
**EMT and stemness**						
Stepwise escalation (0.1 μM to 1 μM) within 6 months	HCC827	Osimertinib	Zeb1 upregulation	JMF3086 (HDAC inhibitor) + osimertinib	WB	Weng et al. (37)
Stepwise dose escalation (0.03 to 1 μmol/L) followed by limiting dilution	H1975	Osimertinib	Zeb1 upregulation with miR-200c downregulation	LY2090314 (GSK-3 inhibitor) + osimertinib	WB, miRNA array	Fukuda et al. (38)
Stepwise method over 6 months	PC9, HCC827	Osimertinib	ANKRD1 overexpression with miR-200 family downregulation	Imatinib + osimertinib	WES, cDNA microarray	Takahashi et al. (39)
Stepwise-dose escalation (500 nm to 1.5 μM) followed by single-cell dilution	H1975	Osimertinib	Downregulation of SQSTM1/p62 and up regulation of LC3	–	WB	Verusingam et al. (40)
Mesenchymal-resistant cell line derived from biopsies of NSCLC patients who progressed on 3rd-generation EGFR TKIs	–	EGF816	Hight expression of FGFR1 and FGF2	BGJ39 (FGFR1/2/3 inhibitor) with EGF816 (nazartinib)	Whole-genome CRISPR screening	Crystal et al. (41); Raoof et al. (42)
Exposure to increasing doses	PC9	Osimertinib	HES1 upregulation ALDH1A1 upregulation	–	WB	Codony-Servat et al. (43)
**Apoptosis modulators**						
Gradually increasing concentrations (10 nM to 500 nM) for approximately 6 months	-PC9; Gefitinib-resistantT790 M+ PC9; HCC827	Osimertinib	Bim downregulation with Mcl-1 upregulation	MEK inhibitors (PD0325901; AZD6244; GSK1120212) + osimertinib HDAC inhibitors (SAHA and LBH589) + osimertinib	WB	Shi et al. (44); Zang et al. (45)
Escalating dose exposure (20 nM to 5 μM) for 12–16 weeks followed by single-cell cloning for 12–16 weeks	H1975	AC0010 cross-resist to rociletinib and osimertinib.	BCL-2 upregulation	ABT263 (navitoclax) + AC0010	RNA sequencing, WB	Xu et al. (27)
Stepwise increased concentration (5 μM to 15 μM) over 11 months	H1975	Osimertinib	BCL-2 upregulation	BCL- 2 inhibitors (ABT263/ABT199) + osimertinib	WB, qPCR	Liu et al. (46)
**NF-KB**						
Exposure to escalating concentrations up to 1 μM for 8 to 10 months	H1975	CNX-2006 cross-resist to rociletinib	Overexpression of p105 and of p50	TPCA-1 + CNX-2006 Bortezomib + of CNX-2006 BEZ-235 + of CNX-2006	WB, phospho-kinase array	Galvani et al. (47)
Gradually increasing concentrations: -from 30 nM to 4 µM, for 10 months -from 200 nM up to 4 µM	Gefitinib-resistant PC9 H1975	Rociletinib	Overexpression of p50, p65, IKKα/β and KBα	-Rociletinib + TPCA-1 -Rociletinib + metformin	WB	Pan et al. (48)
Escalating dose exposure (20 nM to 5 μM) for 12–16 weeks followed by single-cell cloning for 12–16 weeks	H1975	AC0010	NFKB1 upregulation	–	RNA sequencing, WB	Xu et al. (27)
**Other mechanism**						
Stepwise dose escalation (50 nM to 1 μM)	PC9, HCC827, H1975, and HCC4006	Rociletinib or osimertinib	AURKA activation with TPX2 overexpression	Alisertib + osimertinib	Drug screening, WB	Shah et al. (49)
Increased concentrations (5 nM to 1.5 μM) over 22 weeks	H1975	Osimertinib	Upregulation of CDK4, CDK6 and CCND1 and hyperphosphorylation of Rb	Palbociclib + osimertinib	Cell cycle analysis, qPCR, WB	Qin et al. (50)
–	HCC827	Osimertinib	IRE1α upregulation	STF-083010 (IRE1α inhibitor)	WB	Tang et al. (51)

## Epigenetics in EGFR-TKIs Resistance in NSCLC

Resistance to EGFR-TKIs may be related to the presence of preexisting drug-resistant subclones that will be selected under treatment pressure (52) or to the expansion of persistent cells (with or without an acquired resistance mechanism) after the initial response to targeted therapy (53–55). The question that arises is how, in the absence of a genetic mechanism triggering the development of resistance, persister cells manage to escape EGFR-TKI therapy. Previous studies have suggested that entering into a drug-tolerant (DT) persister state is an alternative strategy towards acquiring resistance. It has been reported that small cell populations undergo non-genetic adaptations that allow survival in the presence of the drug from which a fraction of cells can grow into the drug (53, 56). The first attempt to characterize the drug-tolerant cells was reported in 2010 by Sharma et al.; DT persister PC9 cells generated by lethal exposure to erlotinib showed upregulation of histone demethylase KDM5A associated with impaired histone deacetylase (HDAC) activity. Interestingly, the cells returned to spontaneous sensitivity after drug withdrawal (57). Subsequent studies identified other targets related to persister DT cells including IGF1-R (58), and Axl (59), in addition to modulation of apoptosis involving Mcl-1 (60) and Aurora kinases (61). In addition to this, sensitivity to 3rd G EGFR-TKIs had been restored in resistant cell lines generated after drug withdrawal (47, 49), suggesting a non-genetic adaptation. Taken together, these studies indicate a potential role for epigenetics in the adaptation persister drug-tolerant cells. Epigenetic modulations are changes that affect cellular phenotype without affecting the DNA sequence. These changes include DNA methylation, post-translational modifications of histones, and small and long non-coding RNA sequences, all of which may be reversible and heritable modifications. While the mechanisms of genetic resistance to 3rd G EGFR-TKIs, in particular osimertinib, are widely investigated in clinical and preclinical studies, the epigenetic involvement is not well characterized and remains poorly understood. Nevertheless, some published data show a strong epigenetic involvement in this phenomenon and invite further investigations. First, the methylcytosine dioxygenase TET2 and the methyltransferase DNMT3A appeared in the mutational profile of NSCLC patients on post-osimertinib therapy (62). Second, HDAC inhibitors have shown synergy with osimertinib in reversing epithelial–mesenchymal transition (EMT)-related resistance linked to stemness, in preclinical models (37, 38). In addition, analysis of circular microRNAs (crmiR) in established osimertinib-resistant cell lines revealed 16,000 differentially expressed crmiRs compared to non-resistant cells (63). MicroRNAs such as the miR-200 family have previously been shown to play a role in acquired resistance to osimertinib (39). Finally, recently, long non-coding RNAs (lncRNAs), CRNDE and DGCR5, have been reported to induce resistance to afatinib and osimertinib *via* downregulation of eIF4A (64).

Notably, a recent report showed that the emergence of EGFR inhibitor resistance in NSCLC may also be nonheritable and attributed to stochastic variations (65).

## Preclinical Models for Acquired Resistance to 3rd G EGFR-TKIs

### EGFR-Dependent Mechanisms

The mechanisms of on-target EGFR resistance consist of genetic alterations in EGFR occurring during progression under 3rd G EGFR-TKIs. In clinical studies, EGFR-dependent resistance is related to additional somatic EGFR mutations and to gene amplification (9, 27). EGFR point mutations occur in the kinase domain and affect the osimertinib covalent binding residue (C797S/G, exon 20), the EGFR solvent-front (G796S/R, exon 20), the hinge region (L792H/F), and residues inducing steric interaction (L718Q/V, G719C/S/A and G724S, exon 18) (62, 66, 67).

In preclinical models, EGFR amplification was reported in an established rociletinib-resistant cell line (**Table 1**) and sensitivity to rociletinib was restored by cetuximab, a specific anti-EGFR monoclonal antibody, or by afatinib (20). Somatic EGFR mutations, however, have not been identified, which could be explained by the efficacy of 3rd G EGFR-TKIs in inhibiting EGFR protein. Thus, to better understand the involvement of EGFR aberrations in the induction of resistance, studies have been limited to ectopic overexpression of the wild-type (25) or mutated protein or to site-directed mutagenesis replicating mutations described in relapsed patients. The C797S mutation, engineered with a deletion within exon 19 in Ba/F3 cells, conferred significant resistance against osimertinib compared to other EGFR variants such as L718V, L792F/H, and G724S. However, when associated with L858R, C797S/G and L718Q/V conferred comparable resistance, which was greater than in a Del19 background. L792F/H, in contrast, induced significantly less resistance with L858R (68). This indicates that the initial activating mutation may play a role in the potency of resistance to osimertinib. Consistent with this finding, it was reported in the FLAURA study that patients with the L858R mutation have a worse prognosis than those with Del19 (7). Importantly, as observed in the clinic (69–72), earlier-generation EGFR-TKIs may be effective against osimertinib resistance; this may depend not only on the position of the mutation but also on its allelic context. Afatinib inhibited EGFR phosphorylation and cell growth in osimertinib-resistant Ba/F3 cells that exogenously express the G724S mutation, alone or with Del19 (67). The S724 variant induced conformational changes that are incompatible with EGFR-TKIs 3rd G and 1st G binding but not with 2nd G (67). Ba/F3 cells expressing EGFR^L858R/C797S^ by N-ethyl-N-nitrosurea mutagenesis were found to be sensitive to gefitinib (73). A recent study showed that sensitivity and response to EGFR-TKIs are also heterogeneous within the same EGFR exon and proposes a new classification rather based on the structure function of the mutation to determine potential future therapeutic approaches (74).

In clinical studies, emergence of the EGFR^C797X^ mutation is the most common mechanism of resistance to EGFR-dependent osimertinib regardless of treatment line. It was detected in 15% of patients progressing to second-line osimertinib therapy (75) and in only 7% of disease progression when osimertinib is administrated in first-line therapy (76). C797S occurred more frequently in association with Del19 than with L858R mutation (77, 78). Otherwise, C797S was observed in less than 3% of cases in rociletinib-resistant patients (25) and was not observed in patients who progressed after AC0010 treatment (79), suggesting that the resistance mechanism might be drug dependent.

### EGFR-Independent Mechanisms

**Figure 1:**
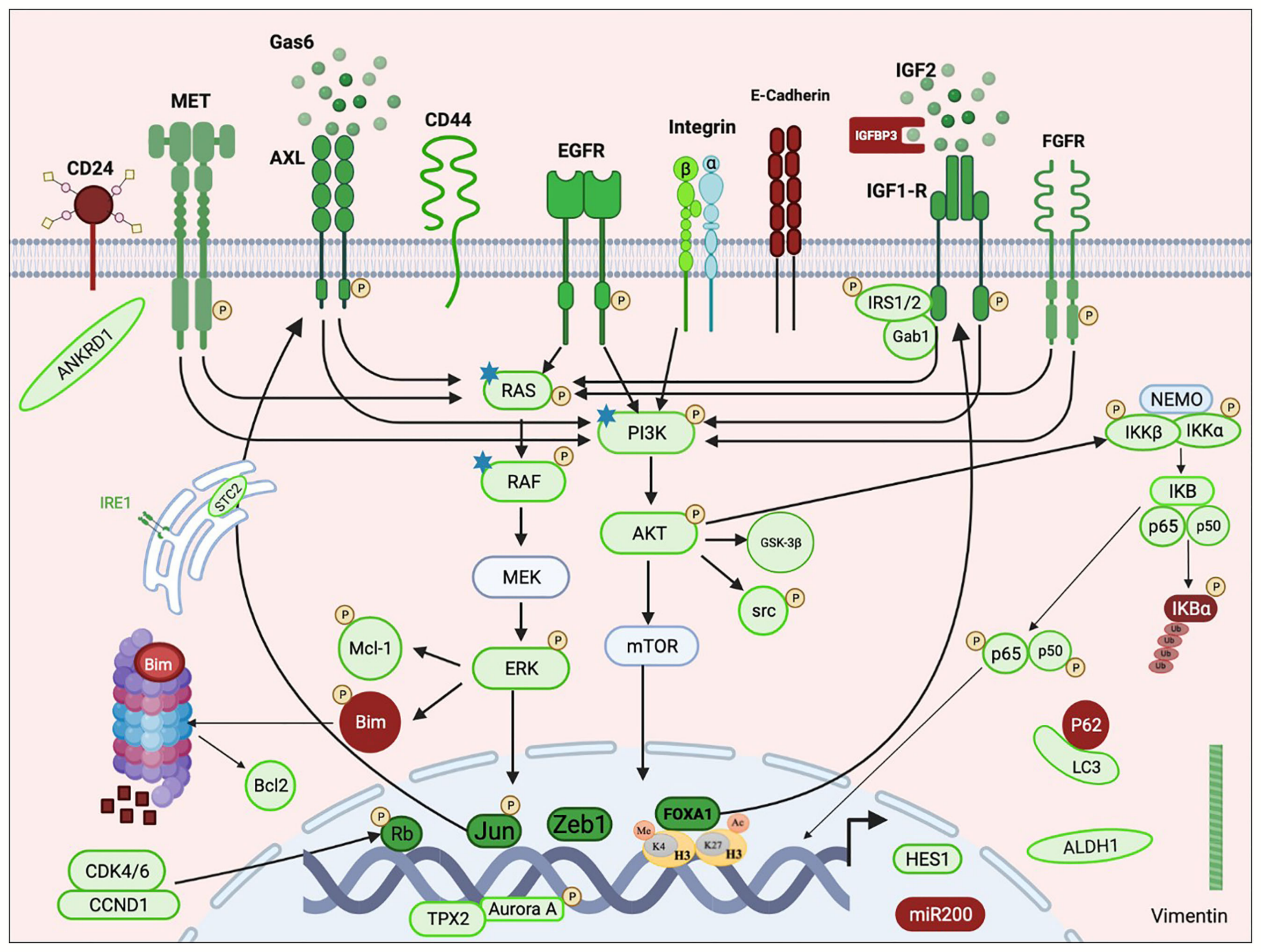
Schematic representation of an overview of pathways implicated in resistance emergence to third-generation EGFR-TKIs. Mechanisms of resistance to third-generation EGFR-TKIs include aberrant activation of receptor tyrosine kinases (MET, AXL, IGF1-R, FGFR, and EGFR) and/or the downstream pathways (PI3K/AKT, RAS/MAPK, and NFKB) and histological transformation. RTK activation is due to overexpression of the protein with or without copy number gain, through its transactivation involving transcription factors (e.g., Jun and FOXA1) or consequently to the overexpression of its specific ligand (e.g., IGF2 and GAS6). Activation of the downstream cascades can also be due to somatic mutations (e.g., RAS, RAF, and PI3K). Histological transformations consist of EMT and EMT-related stemness features including downregulation of E-cadherin and miR200 family, upregulation of Vimentin, Zeb1 and ANKRD1, enrichment in CD44^hight^/CD24^low^ and ALDHA1^hight^ populations, HES1 overexpression, and autophagy activity. Resistance also required apoptosis modulation through Bim degradation and Bcl-1 upregulation. Non-classified resistance mechanisms include activation of AURKA and its coactivator TPX2 and upregulation of CDK4/6 and IRE1. Green color indicates activation or overexpression; red indicates down-regulation. P: phosphorylation. Star: point mutation. This figure was created using the free version of the Biorender website.

Resistance to osimertinib mediated by EGFR-independent mechanisms can be acquired through activation of alternative bypass pathways, aberrant downstream signaling or histologic transformation. **Figure 1** illustrates the involved pathways described in the review.

### MAPK and PI3K Pathways Implication

MAPK and AKT are common downstream modulators of receptor tyrosine kinases (RTKs). Activation of the MAPK pathway *via* ERK activation is a common feature of nearly all preclinical models of resistance to 3rd G EGFR-TKIs. However, alterations of other upstream effectors have also been identified as a driving event in the occurrence of resistance. Copy number gains of MAPK1, CRKL, NRAS, and KRAS as well as single mutations in NRAS (G12V/R, Q61K and E63K) were identified in resistant populations established from PC9 and H1975 cells after chronic exposure to osimertinib or WZ4002 (**Table 1**). Combination with MEK inhibitor Selumetinib prevented the emergence of resistant PC9 cells after 34 days at which time resistant cells appeared in the presence of osimertinib alone, and delayed resistance in H1975 from 17 days in the presence of osimertinib alone to 40 days in the presence of selumetinib. Interestingly, combination therapy induced regression in osimertinib-resistant tumors in transgenic mice carrying EGFR^L858R/T790M^ (21). NRAS amplification was also reported, in a subsequent study, in naquotinib-resistant PC9 cells harboring the T790M mutation (**Table 1**). Either selumetinib or trametinib, a second MEK inhibitor, resensitized naquotinib-resistant cells (22). Single KRAS mutations have also been described in resistance to 3rd G EGFR-TKIs. First, to assess the relevance of KRAS^G12S^ identified in a patient with acquired osimertinib resistance, Ortiz-Cuaran et al. showed that exogenous expression of KRAS^G12S^ in PC9 and HCC827 cells reduced sensitivity to osimertinib and rociletinib, indicating that expression of an activated KRAS allele is sufficient to drive resistance to 3rd-generation TKI (10). In a second study, KRAS^G13D^ mutation was reported as a potential resistance mechanism in osimertinib-resistant PC9 cells (20) (**Table 1**). Another variant, HRAS^G13R^, in association with increased MET expression was reported in osimertinib-resistant PC9 cells (23) (**Table 1**). Moreover, BRAF^G469A^ mutation occurred in osimertinib-resistant PC9 clones (**Table 1**). Similar to the models mentioned, dual therapy using selumetinib or trametinib with osimertinib was effective in overcoming resistance and enhancing cell death in mutated cells (24). To our knowledge, no NRAS alteration has been reported in clinical studies. Alterations in KRAS, however, are widely reported in clinical data. The KRAS^G12S^ mutation was identified in the lymph node biopsy, collected after relapse of osimertinib treatment, but not in plasma sample. Interestingly, EGFR^C797S^ variant, which in turn appeared after relapse, had been found in the patient’s plasma but not in the lymph node biopsy, indicating that different resistance mechanisms may develop at the same time (10). In another study, the KRAS^G12D^ mutation detected in patient’s plasma relapsing on first-line osimertinib therapy was associated with the CTNNB1^S37F^ mutation. Notably, the initial Del19 has not been detected in the post-therapy plasma sample (11). Clearance of the Del19 subpopulation may be due to different sources of the pre- and post-therapy samples or to selection of EGFR^WT^ cells during the development of resistance. The KRAS^Q61R^ variant has also been reported in the occurrence of osimertinib resistance (78). KRAS G12A, Q61H, and A146T variants were found in patients treated with rociletinib that were not detected in their pre-treatment plasma specimens (25). KRAS amplification has also been involved in osimertinib resistance. Molecular profiling of patients who relapsed on osimertinib therapy showed one case of KRAS/MDM2/CDK4 co-amplification (78). Finally, the BRAF^V600E^ mutation has been reported as a mechanism of resistance to osimertinib treatment, both alone (80) and in combination with MET amplification (81). The variant was also identified in a liquid biopsy sample from a patient undergoing treatment with ASP8273, a Japanese 3rd G EGFR-TKI (82). Combination therapies with 3rd G EGFR-TKIs and MEK inhibitors have been developed for lung cancers with EGFR mutations (NCT02143466).

As with ERK, maintained AKT activation is shown in the majority of preclinical models resistant to 3rd G TKIs and its restoration to normal status often required dual therapy. Shigenari et al. reported Src-AKT pathway activation, through Integrin β1 overexpression, as a resistance mechanism in established osimertinib- and rociletinib-resistant H1975 cells (**Table 1**). Either dasatinib or bosutinib, both Src inhibitors, suppressed Src phosphorylation and restored sensitivity to 3rd G EGFR-TKIs. Finally, to understand if the co-occurring of PIK3CA^G106V^ with CTNNB1^S37F^ mutations observed in a patient that had progressed on rociletinib treatment had a role in acquired resistance, HCC827 cells were engineered with single or both mutations. While PIK3CA^G106V^ expression promoted invasion and migration, CTNNB1^S37F^ activated the wnt/beta-catenin pathway, promoted cellular invasion and suppressed apoptosis in response to rociletinib. Authors suggested a non-redundant cooperation of CTNNB1 with PI3CA alterations to promote tumor metastasis or limit EGFR inhibitor response (83). β-catenin has been shown to play an important role in acquired resistance to EGFR-TKIs and EMT in NSCLC cells (84, 85).

In patients, PIK3CA mutations, including E542K, E545K, and E418K, are frequently observed in resistance to 3rd G EGFR-TKIs (25, 62, 78, 83). They accounted for 7% of the resistance mechanisms to first-line osimertinib therapy (76).

## Alternative Receptor Tyrosine Kinase Activation

### MET

Activation of C-mesenchymal–epithelial transition factor (c-MET) appears to be a common resistance mechanism to third-generation EGFR-TKIs, and has been found to be associated with resistance to osimertinib, to rociletinib (10), and to AC0010 (86). In preclinical studies, MET activation is due to gene copy gain or protein overexpression. No MET point mutations have been reported as observed in patients. In an preclinical, *in vivo*, model, rociletinib-resistant tumors were collected from mice bearing PC9 tumors (**Table 1**). Genomic and biochemical analysis revealed MET amplification and activation as the only mechanism of acquired resistance. The combination of rociletinib with crizotinib, a kinase inhibitor with multiple targets including MET, reduced significantly the viability of cell lines derived from rociletinib-resistant tumors. Dual therapy effectively decreased growth on mice of patient-derived xenograft tumors harboring L858R and MET copy gain (25). In a second original model, established by Wanhong et al., AC0010-resistant H1975 cells were generated in two steps first, *in vivo*, and then after selection, *in vitro* (**Table 1**). The derived cells were cross-resistant to EGFR-TKIs of other generations and showed upregulation of MET. Consistent with the above mentioned, double inhibition of EGFR and MET with AC0010 and crizotinib, respectively, prevented colony formation and suppressed MET activation in resistant cell lines and reduced significantly tumor growth in xenograft, compared with single therapy (86). MET activation has also been reported in cell line-based models generated by exposure to progressively increasing concentrations of 3rd G EGFR-TKI. Increased MET copy number was observed in osimertinib-resistant HCC827 cells (**Table 1**), which were also cross-resistant to rociletinib and the 1st-generation EGFR-TKI, erlotinib. MET inhibitors such as crizotinib, ARQ179, or SGX523 sensitized osimertinib-resistant cells (26). In a second model, increased MET phosphorylation was reported to be responsible for acquired resistance in naquotinib-resistant PC9 cells (**Table 1**). Interestingly, dual therapy by naquotinib with crizotinib or SGX-523 had a limited effect on bulk resistant cells, while drastically reducing the proliferation of monoclonal resistant cells, suggesting that heterogeneity may underlie the resistance to a specific TKI target. Notably, the one clone that overexpressed MET protein showed an increase in MET copy number, which was not observed in the resistant parental cells arguing for clonal evolution in the development of resistance. In addition, naquotinib administrated with crizotinib induced robust regression in mice bearing monoclonal resistant cell tumors without apparent cytotoxicity (22).

In clinical studies, MET copy number gains are the most common alternative bypass mechanisms for osimertinib resistance, regardless of treatment line (76, 78). In a recent report, MET amplification was found in 66% (*n* = 6/9) of patients treated with first-line osimertinib (12). Besides osimertinib, MET amplification has also been reported in tumor biopsies from patients with lung adenocarcinoma who developed resistance to rociletinib (10). Point mutations, such as MET P97Q, I865F, and H1094Y, have also been identified in patients with lung cancer progressing on osimertinib (62, 77). The efficacy of MET inhibitors alone or in combined therapy with 3rd G EGFR-TKIs has been reported in clinical studies (87, 88). Recently, the feasibility of combining osimertinib with savolitinib, a potent and selective MET inhibitor, has been tested in clinical trials (89).

### AXL

In NSCLC, it has been reported that anexelekto (AXL) plays a role in resistance to many anti-cancer drugs including EGFR inhibitors (90). Indeed, Tanguichi et al. showed that PC9 cells do not have basal AXL activity as it is the case for MET and HER3 and that AXL phosphorylation appears shortly (4 h) after exposure to osimertinib and increases throughout the exposure period. They also observed a concomitant increase in MET, HER3, and EGFR phosphorylation, suggesting that AXL activation may accelerate the emergence of tolerant cells (59). In addition, drug-tolerant cells isolated from PC9, HCC4011, or H1975, 9 days after high-dose osimertinib treatment (3 μmol/L) expressed a higher level of AXL than parental cells and were highly sensitive to the AXL inhibitor, ONO-7475, in contrast to parental cells (28). Moreover, initial combined treatment with osimertinib and ONO-7475 had more effective effect on tumor regression PC9-derived xenograft when used as the initial treatment than as an alternative therapy once resistance to osimertinib developed (28). Since PC9 cells are enriched in AXL, this indicates that AXL expression level may be a predictor of response to osimertinib. Moreover, primary PE2988 cells, established from the pleural effusion of a patient who developed resistance to osimertinib showed high level of total and phosphorylated AXL and of stanniocalcin (STC2) and responded to the combination of AXL inhibitors and osimertinib (29). STC2 is involved in EGFR-TKIs resistance and was found upregulated in established gefitinib-resistant PC9 and osimertinib-resistant H1975 cells (**Table 1**). Indeed, exogenous overexpression of STC2 activated AXL and increased c-jun level and phosphorylation. In fact, c-jun forms with c-Fos the transcription factor, activation protein-1 (AP-1), which binds to AXL promoter (91), suggesting the involvement of the STC2-JUN-AXL axis in EGFR-TKI resistance (29, 92). Another mechanism of resistance to osimertinib and gefitinib, involving AXL, was described by Kim et al. in osimertinib-resistant HCC827 cells (**Table 1**). The model showed increased expression level of GAS6, a ligand of AXL, and prolonged protein degradation rates in parallel with AXL overexpression. Thus, YD-mediated AXL degradation synergized with osimertinib to restore osimertinib sensitivity *in vitro* and *in vivo* (30). AXL activation was identified as the mechanism of resistance in different established osimertinib-resistant cell lines, despite different EGFR mutational profiles (**Table 1**). It has been observed alone, with an EMT phenotype, or with MET amplification (31–33). Cabozantinib, a multiple TKI including AXL (31), or CB469, a dual inhibitor of MET and AXL, with osimertinib (32) overcame resistance. Notably, resistant clones generated with AXL upregulation lost the T790M subpopulation and some the Del19 population, indicating that clonal evolution leads to heterogeneity in resistance mechanisms. In testing a library of drugs, foretinib, a type II inhibitor targeting a panel of RTKs including MET and AXL, showed the lowest IC_50_ in resistant cell lines, which were T790M-negative (33), indicating that clonal heterogeneity is very likely to impair the efficacy of targeted therapy.

In clinical data, high AXL expression was associated with low RR to osimertinib in patients with EGFR-mutated NSCLC (59). Furthermore, PFS and ORR were inversely correlated with AXL mRNA expression in patients with EGFR-mutated NSCLC (93). Results of phase 1 clinical trials to assess the safety and tolerability of DS-1205c, a specific AXL inhibitor, when combined with osimertinib in metastatic or unresectable subjects with EGFR-mutant NSCLC (NCT03255083) are not yet published.

### IGF1-R

Insulin-like growth factor receptor (IGF-R) activation is involved in EGFR-TKIs resistance in NSCLC cell lines (94) and patients (95). Regarding its implication in resistance to 3rd G EGFR-TKIs, it was shown that drug-tolerant cells obtained 72 h after osimertinib treatment expressed elevated levels of total and phosphorylated IGF1-R without changes in the expression of its ligands, IGF1 and IGF2. This activation in the presence of osimertinib was due to epigenetic activation of its own transcription, mediated by the transcription factor FOX1. Moreover, osimertinib treatment enhanced the association of IGF1-R with its adaptor proteins Gab1 and IRS1, thereby promoting cell survival (58). Indeed, WZ4002-resistant PC9 cells (**Table 1**) showed activated IGF1-R associated with IGFBP3 downregulation. Chemical inhibition of IGF1-R with AG-1024 or the blocking monoclonal anti-IGF1/2 antibody, BI836845, restored sensitivity to WZ4002, *in vitro*, and in xenograft mice (34). Loss of IGFBP3 was shown to induce activation of IGF1-R signaling and enhance resistance to WZ4002 in gefitinib-resistance cell line. Reciprocally, addition of recombinant IGFBP3 was sufficient to restore sensitivity to the 3rd G EGFR-TKI (96). In osimertinib-resistant cell line models, IGF1-R activation was observed in the presence (35) or absence of T790M (**Table 1**). Indeed, a protein phosphorylation array performed in PC9 osimertinib cells (**Table 1**) detected high activity of the “p-Y-IRS1 p-IRS2 bind PI3K” pathway, which is involved in IGF1-R signaling. Interestingly, the pathway was not activated in gefitinib- or erlotinib-resistant PC9, suggesting a mechanism specific to third-generation EGFR inhibitors. In contrast to the above models, the resistant cells did not show IGFBP3 downregulation but did show increased IGF2 expression. The IGF1-R inhibitor linsitinib overcame osimertinib resistance in resistant cell lines and in the patient-derived KOLK43 cells [established from pleural effusion of a erlotinib- and osimertinib-resistant patient with high IGF1-R phosphorylation (36)]. Increased phosphorylation of IGF1-R was observed, by immunohistochemistry, in the tumor sample of an EGFR‐mutated NSCLC patient who acquired resistance to osimertinib (35).

## Histological Transformation

Histological and phenotypic transformations in preclinical models of resistance to 3rd-generation EGFR-TKIs correspond mainly to epithelial–mesenchymal transition (EMT) and to EMT-related stemness. In contrast to clinical data, transformation into small cell lung cancer has not been reported in preclinical models to date. Features of EMT, including decreased E-cadherin and increased vimentin expression, were reported in the generated osimertinib-resistant cell lines. The phenotype was observed in association with upregulation of the zinc finger transcription factor ZEB1 and the formation of spheroids, a feature of stemness (**Table 1**). Reversal of EMT by dual HDAC and the 3-hydroxy-3-methylglutaryl coenzyme A reductase inhibitor, JMF3086, successfully restored sensitivity to osimertinib (37). In fact, ZEB1 could recruit HDAC1 or DNMT1 to the E-cadherin promoter leading to E-cadherin silencing and EMT induction (97). A similar model (**Table 1**) had shown, in addition, a decrease in microRNA-200c expression (38). In fact, the EMT process is governed by a mutually inhibitory miR-200/ZEB feedback loop (98). Glycogen synthase kinase-3-beta (GSK-3β) inhibitor (LY2090314) that emerged in drug screening with significant inhibition of resistant cell growth, in combination with osimertinib, bypassed resistance by suppressing AKT signaling and restoring apoptosis in resistant cells (38). GSK-3β inhibition has been shown to decrease mesenchymal markers and to reduce the associated properties of cancer stem cells (CSC) in aggressive breast cancer (99). A third preclinical model of osimertinib-resistant cells (**Table 1**) reported that the EMT phenotype and downregulation of the miR-200 family were associated with overexpression of Ankyrin Repeat Domain1 (ANKRD1) (39). Indeed, when upregulated, ZEB1 forms a transactivation complex of ANKRD1 with YAP and JUN (100). Imatinib, by inhibiting ANKRD1 and ZEB1, restored apoptosis in resistant cells by increasing levels of Bcl-2 and cleaved PARP (39). Recently, established osimertinib-resistant H1975 clones (**Table 1**) had exhibited EMT characteristics and autophagy activity by downregulating of p62 and upregulation of LC3 (40). Notably, autophagy has been shown to play an important role in promoting cancer metastasis, and inhibition of autophagy might be an effective treatment strategy for malignant cancer (101). Interestingly, whole-genome CRISPR screening in a resistant mesenchymal cell line established from biopsies of NSCLC patients who progressed on 3rd G EGFR-TKIs (41) identified FGFR1 as the top sensitization target of EGF816-resistant cells. Dual EGFR/FGFR inhibition by combining EGF816 with BGJ398, a selective FGFR1–3 inhibitor, induced mesenchymal cell death but had no effect on patient-derived epithelial cell lines (42). In accordance with this, *in vitro* analysis demonstrated that FGF2 supplementation conferred resistance to osimertinib in EGFR mutant NSCLC cells. The same study reported FGFR amplification in patients after progression on osimertinib (102).

Anticancer drug resistance and EMT have been associated with CSCs. However, there are no suitable CSC markers for NSCLC-associated drug resistance and EMT. Upregulation of aldehyde dehydrogenase ALDH1A1, a widely used cancer stem cell marker, was observed in osimertinib-resistant HCC827 cells with EMT features and MET amplification (31) (**Table 1**). EGFR-TKIs, including osimertinib, have been shown to induce enrichment of ALDH positive subpopulations in EGFR-mutated NSCLC models (103, 104), suggesting that specific dual targeting could overcome this adverse effect. Furthermore, osimertinib-resistant PC9 clones (**Table 1**) showed ALDH1A1 or Hairy and enhancer of split homolog-1 (HES1) overexpression (43). HES1 is a transcriptional factor that plays a critical role in gaining and retaining stemness capacity (105). Clinical studies showed HES1 protein levels increased during relapse and were negatively correlated with PFS in EGFR-mutated patients treated with TKIs including osimertinib (43, 106).

## Apoptosis Modulators

Bcl2-like 11 (BIM) has emerged as a key modulator of EGFR-TKI induced apoptosis. Low levels of *BIM* expression in primary tumors are reported to be associated with shorter PFS in patients treated with EGFR-TKI (107). In preclinical studies, osimertinib-resistant PC9 and HCC827 cells (**Table 1**) showed Bim downregulation and Mcl-1 upregulation in association with ERK activation (44). Bim and Mcl-1 are known to be regulated by ERK (108, 109). MEK inhibitors such as PD0325901, AZD6244, or GSK1120212 suppressed phosphorylation of ERK, Bim, and Mcl-1 in cell lines and effectively decreased the growth of osimertinib-resistant xenografts (44). Alternatively, HDAC inhibitors (SAHA and LBH589) plus osimertinib induced significant growth inhibition of osimertinib-resistant cells and xenografts through Bim stabilization (45). Furthermore, a drug screen performed in gefitinib-resistant cells in which WZ4002 failed to restore Bim expression identified ABT-263 (navitoclax), a dual inhibitor of BCL-XL and BCL-2 at the head of compounds that achieve maximal growth inhibition in combination with WZ4002, suggesting a role for BCL-2 in the occurrence of resistance against 3rd G EGFR-TKIs (55). Indeed, RNA sequencing of AC0010-resistant H1975 cells generated *in vitro* (**Table 1**) revealed an overexpression of BCL-2 (8.6-fold compared to parental cells). Dual therapy with ABT263 and AC0010 enhanced apoptosis in resistant cells and reduced colony formation (86). In another model of osimertinib-resistant H1975 cells (**Table 1**) with BCL-2 upregulation, ABT263 as well as ABT199 (BCL-2 inhibitor) synergized with osimertinib to overcome resistance through downregulation of p21or downregulation of SQSTM1 and Survivin (46). Clinical trials studying oral combination therapy with navitoclax and osimertinib in advanced EGFR-mutant NSCLC with prior TKI treatment have reported an ORR of up to 100% and a median PFS of 16.8 months. However, thrombocytopenia and lymphopenia were the most common adverse events (37%) observed in the study (110). Finally, it was found that C-FLIP knockdown restored osimertinib-induced apoptosis in resistant cells (**Table 1**), suggesting that C-FLIP depletion may be an effective strategy to overcome osimertinib resistance in NSCLC (44, 111). Moreover, silencing of C-FLIP had sensitized EGFR-mutant NSCLC to erlotinib and, conversely, its overexpression rescued EGFR-mutant lung cancer cells from erlotinib treatment, presumably through modulation of NF-κB activity (112).

## NF-KB Pathway

Enhanced nuclear factor binding near the κ light chain gene in *B* cells (NF-κB) signaling activity has been implicated as a possible mechanism of resistance to EGFR-TKIs since patients with EGFR mutations who had developed resistance to erlotinib showed low expression of the NF-κB inhibitor, IκBα (112). Activation of NF‐κB by overexpression of NF-κB1 (p50) and its precursor (p105), without altering the expression level of p65, has been reported as a mechanism of acquired resistance against CNX‐2006, a prototype for rociletinib, in resistant H1975 cells (**Table 1**). Notably, resistant cells showed a variety of differences compared to parental cells, but the involvement of NF‐κB was the most studied. Bortezomib, TPCA-1, or BEZ-235, all inhibitors of the NF‐κB pathway, synergized with CNX‐2006 to inhibit cell growth, but with different efficiencies (47). A similar phenotype was observed in established rociletinib-resistant cells (**Table 1**) that expressed higher levels of p50, p65, phospho-IKKα/β, and phospho-KBα proteins than the parental cells. As in the previous model, combination treatment of rociletinib with TPCA‐1 or with Metformin, which is known to inhibit the NF‐κB activity (113), overcame resistance (48). More recently, NF-κB1 was identified among the top ten upregulated gene in AC0010-resistant H1975 generated *in vitro* (**Table 1**), but no further investigations have been conducted to understand the mechanism involved in acquired resistance (86).

### Others

#### AURKA

Aurora kinase A (AURKA) is a serine/threonine kinase that plays a key role during cell division particularly in the process of chromosome segregation (114). The Aurora kinase inhibitors, barasertib and VX680, were identified at the top of the list of drugs that synergized with osimertinib or rociletinib to reduce the growth of generated resistant cell lines, respectively (**Table 1**). Mechanistically, phosphorylation of AURKA was associated with increasing TPX2 protein level following abolition of its CDH1-dependent degradation due to CDH1 sequestration in the cytosol (49). Moreover, barasertib and tozasertib, a second AURK inhibitor, showed a significant antiproliferative effect on osimertinib-resistant cells with no observed difference in AURK expression level (33). Recently, the importance of AURK inhibition in enhancing BIM- and PUMA-mediated apoptosis upon EGFR-TKI therapy in EGFR-mutated lung cancer cells has been described (61). TPX2 expression was significantly increased in tumor tissue samples obtained from patients with advanced EGFR-mutant NSCLC after erlotinib treatment failure compared with results from pre-treatment samples (49).

A Phase 1/1b clinical trial of AURKA inhibitor, Alisertib, with osimertinib in *EGFR*-mutant stage IV metastatic lung cancer is currently recruiting participants (NCT04085315).

#### CDK4/6

Upregulation of CDK4 and CDK6 together with hyperphosphorylation of Rb have been reported as a mechanism of resistance to osimertinib in H1975-resistant cells (**Table 1**). The combination of palbociclib, a selective and potent inhibitor of CDK4/6, with osimertinib overcomes the resistance (50). Acquired alterations in cell cycle genes, including amplification of CDK4/6, CCND1, CCND2, and CCNE1, account for 10% of the acquired resistance mechanisms detected in patients who relapsed after first-line treatment with osimertinib (76).

#### IRE1

Zheng-Hai Tang et al. suggested an increase in Inositol requiring enzyme 1α (IRE1α) expression as a mechanism of resistance to osimertinib in resistant HCC827established *in vitro* (**Table 1**). Indeed, IRE1α knockdown or STF-083010, an inhibitor of IRE1α, reduces cell viability in resistant cells (51).

## Conclusion and Perspectives

In preclinical studies, resistance to 3rd G EGFR-TKIs is mainly due to genetic alterations that increase activity of receptor tyrosine kinases (such as MET, IGF1-R, and AXL) and downstream signaling cascades (such as RAS/MAPK and PI3K/AKT). Histological transformations are limited to EMT and EMT-related stemness. Multiple mechanisms of resistance could be observed in the same population highlighting the heterogeneity of the process, which may be explained in part by clonal evolution, and suggesting that combination therapies will be required to overcome acquired resistance. At this stage, we cannot conclude which of the mechanisms identified in the *in vitro* or *in vivo* models are the closest to what is described in the clinic because of limited data on *in vivo* studies. In general, the developed models do not really reflect the diversity of mechanisms observed in the clinic. Somatic alterations in EGFR, HER2 amplification, and gene fusions (e.g., ALK, RET, and BRAF fusions) are not identified as mechanisms of resistance to 3rd G EGFR-TKIs in the preclinical models. Nevertheless, the models described highlight the utility of early dual therapy and provide insights into possible combination therapies to optimize treatment lines. They also allow the identification of potential biomarkers in pre-existing resistant cells that will emerge under selective pressures, hence the need to develop new relevant preclinical models. NSCLC organoids derived from primary patient tumors or patient-derived xenograft tumors have been shown to maintain the histologic and tumorigenic properties of the parental cancer cells and reflect the drug responses of the parental tumor (115). Such models as well as murine models of patient-derived xenograft and syngeneic lung cancer may be suitable to further investigate resistance mechanisms that are not identified *in vitro*, such as EGFR mutations, or small cell or squamous cell transformation, while preserving the authenticity of the tumor. They could also allow the anticipation of investigations on new-generation therapeutic strategies.

## Author Contributions

RP conceived and supervised the project direction. EM and GL inquired and collected the literature. EM read the literature, wrote the manuscript, and prepared the figure and table. CR and CM revised the manuscript. RP and HL read and approved the final draft. All authors contributed to the article and approved the submitted version.

## Funding

This work was funded by post-doctoral fellowship from Association pour la recherche contre le cancer (ARC), Ligue contre le cancer Grand Ouest, Institut National de la Santé et de la Recherche Médicale (INSERM).

## Conflict of Interest

RP received a grant from AstraZeneca to conduct a project in the laboratory but it was not used to fund this review.

The remaining authors declare that the research was conducted in the absence of any commercial or financial relationships that could be construed as a potential conflict of interest.

## Publisher’s Note

All claims expressed in this article are solely those of the authors and do not necessarily represent those of their affiliated organizations, or those of the publisher, the editors and the reviewers. Any product that may be evaluated in this article, or claim that may be made by its manufacturer, is not guaranteed or endorsed by the publisher.

## References

[1] Mok TS Wu Y-L Thongprasert S Yang C-H Chu D-T Saijo N. Gefitinib or Carboplatin–Paclitaxel in Pulmonary Adenocarcinoma. N Engl J Med (2009) 361:947–57. doi: 10.1056/NEJMoa0810699 19692680

[2] Sequist LV Yang JC-H Yamamoto N O’Byrne K Hirsh V Mok T. Phase III Study of Afatinib or Cisplatin Plus Pemetrexed in Patients With Metastatic Lung Adenocarcinoma With EGFR Mutations. J Clin Oncol (2013) 31:3327–34. doi: 10.1200/JCO.2012.44.2806 23816960

[3] Kobayashi S Boggon TJ Dayaram T Jänne PA Kocher O Meyerson M. EGFR Mutation and Resistance of Non-Small-Cell Lung Cancer to Gefitinib. N Engl J Med (2005) 352:786–92. doi: 10.1056/NEJMoa044238 15728811

[4] Kosaka T Yatabe Y Endoh H Yoshida K Hida T Tsuboi M. Analysis of Epidermal Growth Factor Receptor Gene Mutation in Patients With Non-Small Cell Lung Cancer and Acquired Resistance to Gefitinib. Clin Cancer Res (2006) 12:5764–9. doi: 10.1158/1078-0432.CCR-06-0714 17020982

[5] Pirker R. Third-Generation Epidermal Growth Factor Receptor Tyrosine Kinase Inhibitors in Advanced Nonsmall Cell Lung Cancer. Curr Opin Oncol (2016) 28:115–21. doi: 10.1097/CCO.0000000000000260 26720671

[6] Barnes TA O’Kane GM Vincent MD Leighl NB. Third-Generation Tyrosine Kinase Inhibitors Targeting Epidermal Growth Factor Receptor Mutations in Non-Small Cell Lung Cancer. Front Oncol (2017) 7:113:113. doi: 10.3389/fonc.2017.00113 28620581 PMC5449484

[7] Soria J-C Ohe Y Vansteenkiste J Reungwetwattana T Chewaskulyong B Lee KH. Osimertinib in Untreated *EGFR* -Mutated Advanced Non–Small-Cell Lung Cancer. N Engl J Med (2018) 378:113–25. doi: 10.1056/NEJMoa1713137 29151359

[8] Zhang Q Zhang X-C Yang J-J Yang Z-F Bai Y Su J. EGFR L792H and G796R: Two Novel Mutations Mediating Resistance to the Third-Generation EGFR Tyrosine Kinase Inhibitor Osimertinib. J Thorac Oncol (2018) 13:1415–21. doi: 10.1016/j.jtho.2018.05.024 29857056

[9] Thress KS Paweletz CP Felip E Cho BC Stetson D Dougherty B. Acquired EGFR C797S Mutation Mediates Resistance to AZD9291 in Non–Small Cell Lung Cancer Harboring EGFR T790M. Nat Med (2015) 21:560–2. doi: 10.1038/nm.3854 PMC4771182 25939061

[10] Ortiz-Cuaran S Scheffler M Plenker D Dahmen L Scheel AH Fernandez-Cuesta L. Heterogeneous Mechanisms of Primary and Acquired Resistance to Third-Generation EGFR Inhibitors. Clin Cancer Res (2016) 22:4837–47. doi: 10.1158/1078-0432.CCR-15-1915 27252416

[11] Ramalingam SS Yang JC-H Lee CK Kurata T Kim D-W John T. Osimertinib As First-Line Treatment of *EGFR* Mutation–Positive Advanced Non–Small-Cell Lung Cancer. JCO (2018) 36:841–9. doi: 10.1200/JCO.2017.74.7576 28841389

[12] Roper N Brown A-L Wei JS Pack S Trindade C Kim C. Clonal Evolution and Heterogeneity of Osimertinib Acquired Resistance Mechanisms in EGFR Mutant Lung Cancer. Cell Rep Med (2020) 1:100007. doi: 10.1016/j.xcrm.2020.100007 32483558 PMC7263628

[13] Leonetti A Sharma S Minari R Perego P Giovannetti E Tiseo M. Resistance Mechanisms to Osimertinib in EGFR-Mutated Non-Small Cell Lung Cancer. Br J Cancer (2019) 121:725–37. doi: 10.1038/s41416-019-0573-8 PMC6889286 31564718

[14] Schmid S Li JJN Leighl NB. Mechanisms of Osimertinib Resistance and Emerging Treatment Options. Lung Cancer (2020) 147:123–9. doi: 10.1016/j.lungcan.2020.07.014 32693293

[15] Lazzari C Gregorc V Karachaliou N Rosell R Santarpia M. Mechanisms of Resistance to Osimertinib. J Thorac Dis (2020) 12:2851–8. doi: 10.21037/jtd.2019.08.30 PMC7330330 32642198

[16] Piper-Vallillo AJ Sequist LV Piotrowska Z. Emerging Treatment Paradigms for EGFR-Mutant Lung Cancers Progressing on Osimertinib: A Review. JCO (2020) 38:2926–36. doi: 10.1200/JCO.19.03123 32552277

[17] Passaro A Jänne PA Mok T Peters S. Overcoming Therapy Resistance in EGFR-Mutant Lung Cancer. Nat Cancer (2021) 2:377–91. doi: 10.1038/s43018-021-00195-8 35122001

[18] Ricordel C Friboulet L Facchinetti F Soria J-C. Molecular Mechanisms of Acquired Resistance to Third-Generation EGFR-TKIs in EGFR T790M-Mutant Lung Cancer. Ann Oncol (2018) 29:i28–37. doi: 10.1093/annonc/mdx705 29462256

[19] Ohara S Suda K Mitsudomi T. Cell Line Models for Acquired Resistance to First-Line Osimertinib in Lung Cancers—Applications and Limitations. Cells (2021) 10:354. doi: 10.3390/cells10020354 33572269 PMC7915563

[20] Nukaga S Yasuda H Tsuchihara K Hamamoto J Masuzawa K Kawada I. Amplification of EGFR Wild-Type Alleles in Non–Small Cell Lung Cancer Cells Confers Acquired Resistance to Mutation-Selective EGFR Tyrosine Kinase Inhibitors. Cancer Res (2017) 77:2078–89. doi: 10.1158/0008-5472.CAN-16-2359 28202511

[21] Eberlein CA Stetson D Markovets AA Al-Kadhimi KJ Lai Z Fisher PR. Acquired Resistance to the Mutant-Selective EGFR Inhibitor AZD9291 Is Associated With Increased Dependence on RAS Signaling in Preclinical Models. Cancer Res (2015) 75:2489–500. doi: 10.1158/0008-5472.CAN-14-3167 PMC4605607 25870145

[22] Ninomiya K Ohashi K Makimoto G Tomida S Higo H Kayatani H. MET or NRAS Amplification is an Acquired Resistance Mechanism to the Third-Generation EGFR Inhibitor Naquotinib. Sci Rep (2018) 8:1955. doi: 10.1038/s41598-018-20326-z 29386539 PMC5792548

[23] Ku BM Choi MK Sun J-M Lee S-H Ahn JS Park K. Acquired Resistance to AZD9291 as an Upfront Treatment Is Dependent on ERK Signaling in a Preclinical Model. PloS One (2018) 13:e0194730. doi: 10.1371/journal.pone.0194730 29641535 PMC5895014

[24] La Monica S Minari R Cretella D Bonelli M Fumarola C Cavazzoni A. Acquired BRAF G469A Mutation as a Resistance Mechanism to First-Line Osimertinib Treatment in NSCLC Cell Lines Harboring an EGFR Exon 19 Deletion. Targ Oncol (2019) 14:619–26. doi: 10.1007/s11523-019-00669-x 31502118

[25] Chabon JJ Simmons AD Lovejoy AF Esfahani MS Newman AM Haringsma HJ. Circulating Tumour DNA Profiling Reveals Heterogeneity of EGFR Inhibitor Resistance Mechanisms in Lung Cancer Patients. Nat Commun (2016) 7:11815. doi: 10.1038/ncomms11815 27283993 PMC4906406

[26] Shi P Oh Y-T Zhang G Yao W Yue P Li Y. Met Gene Amplification and Protein Hyperactivation Is a Mechanism of Resistance to Both First and Third Generation EGFR Inhibitors in Lung Cancer Treatment. Cancer Lett (2016) 380:494–504. doi: 10.1016/j.canlet.2016.07.021 27450722

[27] Xu H Shen J Xiang J Li H Li B Zhang T. Characterization of Acquired Receptor Tyrosine–Kinase Fusions as Mechanisms of Resistance to EGFR Tyrosine–Kinase Inhibitors. CMAR (2019) 11:6343–51. doi: 10.2147/CMAR.S197337 PMC6628603 31372039

[28] Okura N Nishioka N Yamada T Taniguchi H Tanimura K Katayama Y. ONO-7475, a Novel AXL Inhibitor, Suppresses the Adaptive Resistance to Initial EGFR-TKI Treatment in *EGFR* -Mutated Non–Small Cell Lung Cancer. Clin Cancer Res (2020) 26:2244–56. doi: 10.1158/1078-0432.CCR-19-2321 31953310

[29] Liu Y Tsai M Wu S Chang T Tsai T Gow C. Acquired Resistance to EGFR Tyrosine Kinase Inhibitors Is Mediated by the Reactivation of STC2/JUN/AXL Signaling in Lung Cancer. Int J Cancer (2019) 145:1609–24. doi: 10.1002/ijc.32487 31162839

[30] Kim D Bach D-H Fan Y-H Luu T-T-T Hong J-Y Park HJ. AXL Degradation in Combination With EGFR-TKI can Delay and Overcome Acquired Resistance in Human Non-Small Cell Lung Cancer Cells. Cell Death Dis (2019) 10:361. doi: 10.1038/s41419-019-1601-6 31043587 PMC6494839

[31] Namba K Shien K Takahashi Y Torigoe H Sato H Yoshioka T. Activation of AXL as a Preclinical Acquired Resistance Mechanism Against Osimertinib Treatment in *EGFR* -Mutant Non–Small Cell Lung Cancer Cells. Mol Cancer Res (2019) 17:499–507. doi: 10.1158/1541-7786.MCR-18-0628 30463991

[32] Yang Y-M Jang Y Lee SH Kang B Lim SM. AXL/MET Dual Inhibitor, CB469, has Activity in Non-Small Cell Lung Cancer With Acquired Resistance to EGFR TKI With AXL or MET Activation. Lung Cancer (2020) 146:70–7. doi: 10.1016/j.lungcan.2020.05.031 32521387

[33] Bertran-Alamillo J Cattan V Schoumacher M Codony-Servat J Giménez-Capitán A Cantero F. AURKB as a Target in Non-Small Cell Lung Cancer With Acquired Resistance to Anti-EGFR Therapy. Nat Commun (2019) 10:1812. doi: 10.1038/s41467-019-09734-5 31000705 PMC6472415

[34] Park JH Choi YJ Kim SY Lee J-E Sung KJ Park S. Activation of the IGF1R Pathway Potentially Mediates Acquired Resistance to Mutant-Selective 3rd-Generation EGF Receptor Tyrosine Kinase Inhibitors in Advanced Non-Small Cell Lung Cancer. Oncotarget (2016) 7:22005–15. doi: 10.18632/oncotarget.8013 PMC5008340 26980747

[35] Hayakawa D Takahashi F Mitsuishi Y Tajima K Hidayat M Winardi W. Activation of Insulin-Like Growth Factor-1 Receptor Confers Acquired Resistance to Osimertinib in Non-Small Cell Lung Cancer With *EGFR* T790M Mutation. Thorac Cancer (2020) 11:140–9. doi: 10.1111/1759-7714.13255 PMC6938756 31758670

[36] Manabe T Yasuda H Terai H Kagiwada H Hamamoto J Ebisudani T. IGF2 Autocrine-Mediated IGF1R Activation Is a Clinically Relevant Mechanism of Osimertinib Resistance in Lung Cancer. Mol Cancer Res (2020) 18:549–59. doi: 10.1158/1541-7786.MCR-19-0956 31941753

[37] Weng C-H Chen L-Y Lin Y-C Shih J-Y Lin Y-C Tseng R-Y. Epithelial-Mesenchymal Transition (EMT) Beyond EGFR Mutations Per Se Is a Common Mechanism for Acquired Resistance to EGFR TKI. Oncogene (2019) 38:455–68. doi: 10.1038/s41388-018-0454-2 30111817

[38] Fukuda K Takeuchi S Arai S Kita K Tanimoto A Nishiyama A. Glycogen Synthase Kinase-3 Inhibition Overcomes Epithelial-Mesenchymal Transition-Associated Resistance to Osimertinib in *EGFR* -Mutant Lung Cancer. Cancer Sci (2020) 111:2374–84. doi: 10.1111/cas.14454 PMC7385349 32391602

[39] Takahashi A Seike M Chiba M Takahashi S Nakamichi S Matsumoto M. Ankyrin Repeat Domain 1 Overexpression Is Associated With Common Resistance to Afatinib and Osimertinib in EGFR-Mutant Lung Cancer. Sci Rep (2018) 8:14896. doi: 10.1038/s41598-018-33190-8 30291293 PMC6173712

[40] Verusingam ND Chen Y-C Lin H-F Liu C-Y Lee M-C Lu K-H. Generation of Osimertinib-Resistant Cells From Epidermal Growth Factor Receptor L858R/T790M Mutant Non-Small Cell Lung Carcinoma Cell Line. J Chin Med Assoc (2021) 84:248–54. doi: 10.1097/JCMA.0000000000000438 PMC12966177 33009209

[41] Crystal AS Shaw AT Sequist LV Friboulet L Niederst MJ Lockerman EL. Patient-Derived Models of Acquired Resistance can Identify Effective Drug Combinations for Cancer. Science (2014) 346:1480–6. doi: 10.1126/science.1254721 PMC4388482 25394791

[42] Raoof S Mulford IJ Frisco-Cabanos H Nangia V Timonina D Labrot E. Targeting FGFR Overcomes EMT-Mediated Resistance in EGFR Mutant Non-Small Cell Lung Cancer. Oncogene (2019) 38:6399–413. doi: 10.1038/s41388-019-0887-2 PMC6742540 31324888

[43] Codony-Servat J Codony-Servat C Cardona AF Giménez-Capitán A Drozdowskyj A Berenguer J. Cancer Stem Cell Biomarkers in EGFR-Mutation-Positive Non-Small-Cell Lung Cancer. Clin Lung Cancer (2019) 20:167–77. doi: 10.1016/j.cllc.2019.02.005 30885551

[44] Shi P Oh Y-T Deng L Zhang G Qian G Zhang S. Overcoming Acquired Resistance to AZD9291, A Third-Generation EGFR Inhibitor, Through Modulation of MEK/ERK-Dependent Bim and Mcl-1 Degradation. Clin Cancer Res (2017) 23:6567–79. doi: 10.1158/1078-0432.CCR-17-1574 PMC5668147 28765329

[45] Zang H Qian G Zong D Fan S Owonikoko TK Ramalingam SS. Overcoming Acquired Resistance of Epidermal Growth Factor Receptor-Mutant Non–Small Cell Lung Cancer Cells to Osimertinib by Combining Osimertinib With the Histone Deacetylase Inhibitor Panobinostat (LBH589). Cancer (2020) 126:2024–33. doi: 10.1002/cncr.32744 PMC7241261 31999837

[46] Liu Z Gao W. Synergistic Effects of Bcl-2 Inhibitors With AZD9291 on Overcoming the Acquired Resistance of AZD9291 in H1975 Cells. Arch Toxicol (2020) 94:3125–36. doi: 10.1007/s00204-020-02816-0 PMC7423640 32577785

[47] Galvani E Sun J Leon LG Sciarrillo R Narayan RS Tjin Tham Sjin R. NF-κb Drives Acquired Resistance to a Novel Mutant-Selective EGFR Inhibitor. Oncotarget (2015) 6:42717–32. doi: 10.18632/oncotarget.3956 PMC4767465 26015408

[48] Pan Y-H Lin C-Y Lu C-H Li L Wang Y-B Chen H-Y. Metformin Synergistically Enhances the Antitumor Activity of the Third-Generation EGFR-TKI CO-1686 in Lung Cancer Cells Through Suppressing NF-κb Signaling: Pan Et al. Clin Respir J (2018) 12:2642–52. doi: 10.1111/crj.12970 30307719

[49] Shah KN Bhatt R Rotow J Rohrberg J Olivas V Wang VE. Aurora Kinase A Drives the Evolution of Resistance to Third-Generation EGFR Inhibitors in Lung Cancer. Nat Med (2019) 25:111–8. doi: 10.1038/s41591-018-0264-7 PMC6324945 30478424

[50] Qin Q Li X Liang X Zeng L Wang J Sun L. CDK4/6 Inhibitor Palbociclib Overcomes Acquired Resistance to Third-Generation EGFR Inhibitor Osimertinib in Non-Small Cell Lung Cancer (NSCLC). Thorac Cancer (2020) 11:2389–97. doi: 10.1111/1759-7714.13521 PMC7471056 32677256

[51] Tang Z Su M-X Guo X Jiang X-M Jia L Chen X. Increased Expression of IRE1α Associates With the Resistant Mechanism of Osimertinib (AZD9291)-Resistant Non-Small Cell Lung Cancer HCC827/OSIR Cells. ACAMC (2018) 18:550–5. doi: 10.2174/1871520617666170719155517 28730963

[52] Wang J Wang B Chu H Yao Y. Intrinsic Resistance to EGFR Tyrosine Kinase Inhibitors in Advanced Non-Small-Cell Lung Cancer With Activating EGFR Mutations. Oncol Targets Ther (2016) 9:3711–26. doi: 10.2147/OTT.S106399 PMC4922765 27382309

[53] Ramirez M Rajaram S Steininger RJ Osipchuk D Roth MA Morinishi LS. Diverse Drug-Resistance Mechanisms can Emerge From Drug-Tolerant Cancer Persister Cells. Nat Commun (2016) 7:10690. doi: 10.1038/ncomms10690 26891683 PMC4762880

[54] Oxnard GR. The Cellular Origins of Drug Resistance in Cancer. Nat Med (2016) 22:232–4. doi: 10.1038/nm.4058 26937615

[55] Hata AN Niederst MJ Archibald HL Gomez-Caraballo M Siddiqui FM Mulvey HE. Tumor Cells can Follow Distinct Evolutionary Paths to Become Resistant to Epidermal Growth Factor Receptor Inhibition. Nat Med (2016) 22:262–9. doi: 10.1038/nm.4040 PMC4900892 26828195

[56] De Conti G Dias MH Bernards R. Fighting Drug Resistance Through the Targeting of Drug-Tolerant Persister Cells. Cancers (2021) 13:1118. doi: 10.3390/cancers13051118 33807785 PMC7961328

[57] Sharma SV Lee DY Li B Quinlan MP Takahashi F Maheswaran S. A Chromatin-Mediated Reversible Drug-Tolerant State in Cancer Cell Subpopulations. Cell (2010) 141:69–80. doi: 10.1016/j.cell.2010.02.027 20371346 PMC2851638

[58] Wang R Yamada T Kita K Taniguchi H Arai S Fukuda K. Transient IGF-1R Inhibition Combined With Osimertinib Eradicates AXL-Low Expressing EGFR Mutated Lung Cancer. Nat Commun (2020) 11:4607. doi: 10.1038/s41467-020-18442-4 32929081 PMC7490421

[59] Taniguchi H Yamada T Wang R Tanimura K Adachi Y Nishiyama A. AXL Confers Intrinsic Resistance to Osimertinib and Advances the Emergence of Tolerant Cells. Nat Commun (2019) 10:259. doi: 10.1038/s41467-018-08074-0 30651547 PMC6335418

[60] Song K-A Hosono Y Turner C Jacob S Lochmann TL Murakami Y. Increased Synthesis of MCL-1 Protein Underlies Initial Survival of *EGFR* -Mutant Lung Cancer to EGFR Inhibitors and Provides a Novel Drug Target. Clin Cancer Res (2018) 24:5658–72. doi: 10.1158/1078-0432.CCR-18-0304 30087143

[61] Tanaka K Yu HA Yang S Han S Selcuklu SD Kim K. Targeting Aurora B Kinase Prevents and Overcomes Resistance to EGFR Inhibitors in Lung Cancer by Enhancing BIM- and PUMA-Mediated Apoptosis. Cancer Cell (2021) 39:1245–61.e6. doi: 10.1016/j.ccell.2021.07.006 34388376 PMC8440494

[62] Yang Z Yang N Ou Q Xiang Y Jiang T Wu X. Investigating Novel Resistance Mechanisms to Third-Generation EGFR Tyrosine Kinase Inhibitor Osimertinib in Non–Small Cell Lung Cancer Patients. Clin Cancer Res (2018) 24:3097–107. doi: 10.1158/1078-0432.CCR-17-2310 29506987

[63] Chen T Luo J Gu Y Huang J Luo Q Yang Y. Comprehensive Analysis of Circular RNA Profiling in AZD9291-Resistant Non-Small Cell Lung Cancer Cell Lines. Thorac Cancer (2019) 10:930–41. doi: 10.1111/1759-7714.13032 PMC6449233 30883029

[64] Takahashi S Noro R Seike M Zeng C Matsumoto M Yoshikawa A. Long Non-Coding RNA CRNDE Is Involved in Resistance to EGFR Tyrosine Kinase Inhibitor in EGFR-Mutant Lung Cancer *via* Eif4a3/MUC1/EGFR Signaling. IJMS (2021) 22:4005. doi: 10.3390/ijms22084005 33924522 PMC8070547

[65] Hayford CE Tyson DR Robbins CJ Frick PL Quaranta V Harris LA. An *In Vitro* Model of Tumor Heterogeneity Resolves Genetic, Epigenetic, and Stochastic Sources of Cell State Variability. PloS Biol (2021) 19:e3000797. doi: 10.1371/journal.pbio.3000797 34061819 PMC8195356

[66] Klempner S Mehta P Schrock A Ali S Ou S-HI. Cis-Oriented Solvent-Front EGFR G796S Mutation in Tissue and ctDNA in a Patient Progressing on Osimertinib: A Case Report and Review of the Literature. LCTT (2017) 8:241–7. doi: 10.2147/LCTT.S147129 PMC5723122 29255376

[67] Fassunke J Müller F Keul M Michels S Dammert MA Schmitt A. Overcoming EGFRG724S-Mediated Osimertinib Resistance Through Unique Binding Characteristics of Second-Generation EGFR Inhibitors. Nat Commun (2018) 9:4655. doi: 10.1038/s41467-018-07078-0 30405134 PMC6220297

[68] Nishino M Suda K Kobayashi Y Ohara S Fujino T Koga T. Effects of Secondary EGFR Mutations on Resistance Against Upfront Osimertinib in Cells With EGFR-Activating Mutations *In Vitro* . Lung Cancer (2018) 126:149–55. doi: 10.1016/j.lungcan.2018.10.026 30527179

[69] Peled N Roisman LC Miron B Pfeffer R Lanman RB Ilouze M. Subclonal Therapy by Two EGFR TKIs Guided by Sequential Plasma Cell-Free DNA in EGFR -Mutated Lung Cancer. J Thorac Oncol (2017) 12:e81–4. doi: 10.1016/j.jtho.2017.02.023 28286242

[70] Niederst MJ Hu H Mulvey HE Lockerman EL Garcia AR Piotrowska Z. The Allelic Context of the C797S Mutation Acquired Upon Treatment With Third-Generation EGFR Inhibitors Impacts Sensitivity to Subsequent Treatment Strategies. Clin Cancer Res (2015) 21:3924–33. doi: 10.1158/1078-0432.CCR-15-0560 PMC4587765 25964297

[71] Wang Z Yang J-J Huang J Ye J-Y Zhang X-C Tu H-Y. Lung Adenocarcinoma Harboring EGFR T790M and In Trans C797S Responds to Combination Therapy of First- and Third-Generation EGFR TKIs and Shifts Allelic Configuration at Resistance. J Thorac Oncol (2017) 12:1723–7. doi: 10.1016/j.jtho.2017.06.017 28662863

[72] Arulananda S Do H Musafer A Mitchell P Dobrovic A John T. Combination Osimertinib and Gefitinib in C797S and T790M EGFR-Mutated Non-Small Cell Lung Cancer. J Thorac Oncol (2017) 12:1728–32. doi: 10.1016/j.jtho.2017.08.006 28843359

[73] Rangachari D To C Shpilsky JE VanderLaan PA Kobayashi SS Mushajiang M. EGFR-Mutated Lung Cancers Resistant to Osimertinib Through EGFR C797S Respond to First-Generation Reversible EGFR Inhibitors But Eventually Acquire EGFR T790M/C797S in Preclinical Models and Clinical Samples. J Thorac Oncol (2019) 14:1995–2002. doi: 10.1016/j.jtho.2019.07.016 31377341 PMC6823139

[74] Robichaux JP Le X Vijayan RSK Hicks JK Heeke S Elamin YY. Structure-Based Classification Predicts Drug Response in EGFR-Mutant NSCLC. Nature (2021) 597:732–7. doi: 10.1038/s41586-021-03898-1 PMC8481125 34526717

[75] Papadimitrakopoulou VA Wu Y-L Han J-Y Ahn M-J Ramalingam SS John T. Analysis of Resistance Mechanisms to Osimertinib in Patients With EGFR T790M Advanced NSCLC From the AURA3 Study. Ann Oncol (2018) 29:viii741. doi: 10.1093/annonc/mdy424.064

[76] Ramalingam SS Cheng Y Zhou C Ohe Y Imamura F Cho BC. Mechanisms of Acquired Resistance to First-Line Osimertinib: Preliminary Data From the Phase III FLAURA Study. Ann Oncol (2018) 29:viii740. doi: 10.1093/annonc/mdy424.063

[77] Schoenfeld AJ Chan JM Kubota D Sato H Rizvi H Daneshbod Y. Tumor Analyses Reveal Squamous Transformation and Off-Target Alterations As Early Resistance Mechanisms to First-Line Osimertinib in *EGFR* -Mutant Lung Cancer. Clin Cancer Res (2020) 26:2654–63. doi: 10.1158/1078-0432.CCR-19-3563 PMC7448565 31911548

[78] Le X Puri S Negrao MV Nilsson MB Robichaux J Boyle T. Landscape of EGFR-Dependent and -Independent Resistance Mechanisms to Osimertinib and Continuation Therapy Beyond Progression in *EGFR* -Mutant NSCLC. Clin Cancer Res (2018) 24:6195–203. doi: 10.1158/1078-0432.CCR-18-1542 PMC6295279 30228210

[79] Ma Y Zheng X Zhao H Fang W Zhang Y Ge J. First-In-Human Phase I Study of AC0010, a Mutant-Selective EGFR Inhibitor in Non–Small Cell Lung Cancer: Safety, Efficacy, and Potential Mechanism of Resistance. J Thorac Oncol (2018) 13:968–77. doi: 10.1016/j.jtho.2018.03.025 29626621

[80] Ho C-C Liao W-Y Lin C-A Shih J-Y Yu C-J Yang JC-H. Acquired BRAF V600E Mutation as Resistant Mechanism After Treatment With Osimertinib. J Thorac Oncol (2017) 12:567–72. doi: 10.1016/j.jtho.2016.11.2231 27923714

[81] Minari R Bordi P La Monica S Squadrilli A Leonetti A Bottarelli L. Concurrent Acquired BRAF V600E Mutation and MET Amplification as Resistance Mechanism of First-Line Osimertinib Treatment in a Patient With EGFR-Mutated NSCLC. J Thorac Oncol (2018) 13:e89–91. doi: 10.1016/j.jtho.2018.03.013 29596911

[82] Bearz A De Carlo E Doliana R Schiappacassi M. Acquired BRAF V600E Mutation as Resistant Mechanism After Treatment With Third-Generation EGFR Tyrosine Kinase Inhibitor. J Thorac Oncol (2017) 12:e181–2. doi: 10.1016/j.jtho.2017.07.017 29074209

[83] Blakely CM Watkins TBK Wu W Gini B Chabon JJ McCoach CE. Evolution and Clinical Impact of Co-Occurring Genetic Alterations in Advanced-Stage EGFR-Mutant Lung Cancers. Nat Genet (2017) 49:1693–704. doi: 10.1038/ng.3990 PMC5709185 29106415

[84] Fang X Gu P Zhou C Liang A Ren S Liu F. β-Catenin Overexpression Is Associated With Gefitinib Resistance in Non-Small Cell Lung Cancer Cells. Pulm Pharmacol Ther (2014) 28:41–8. doi: 10.1016/j.pupt.2013.05.005 23707949

[85] Togashi Y Hayashi H Terashima M de Velasco MA Sakai K Fujita Y. Inhibition of β-Catenin Enhances the Anticancer Effect of Irreversible EGFR-TKI in EGFR-Mutated Non–Small-Cell Lung Cancer With a T790M Mutation. J Thorac Oncol (2015) 10:93–101. doi: 10.1097/JTO.0000000000000353 25384171

[86] Xu W Tang W Li T Zhang X Sun Y. Overcoming Resistance to AC0010, a Third Generation of EGFR Inhibitor, by Targeting C-MET and BCL-2. Neoplasia (2019) 21:41–51. doi: 10.1016/j.neo.2018.11.004 30504063 PMC6310688

[87] Ou S-HI Cui J Schrock AB Goldberg ME Zhu VW Albacker L. Emergence of Novel and Dominant Acquired EGFR Solvent-Front Mutations at Gly796 (G796S/R) Together With C797S/R and L792F/H Mutations in One EGFR (L858R/T790M) NSCLC Patient Who Progressed on Osimertinib. Lung Cancer (2017) 108:228–31. doi: 10.1016/j.lungcan.2017.04.003 28625641

[88] Sequist LV Han J-Y Ahn M-J Cho BC Yu H Kim S-W. Osimertinib Plus Savolitinib in Patients With EGFR Mutation-Positive, MET-Amplified, Non-Small-Cell Lung Cancer After Progression on EGFR Tyrosine Kinase Inhibitors: Interim Results From a Multicentre, Open-Label, Phase 1b Study. Lancet Oncol (2020) 21:373–86. doi: 10.1016/S1470-2045(19)30785-5 32027846

[89] Oxnard GR Yang JC-H Yu H Kim S-W Saka H Horn L. TATTON: A Multi-Arm, Phase Ib Trial of Osimertinib Combined With Selumetinib, Savolitinib, or Durvalumab in EGFR-Mutant Lung Cancer. Ann Oncol (2020) 31:507–16. doi: 10.1016/j.annonc.2020.01.013 32139298

[90] Zhang Z Lee JC Lin L Olivas V Au V LaFramboise T. Activation of the AXL Kinase Causes Resistance to EGFR-Targeted Therapy in Lung Cancer. Nat Genet (2012) 44:852–60. doi: 10.1038/ng.2330 PMC3408577 22751098

[91] Mudduluru G Leupold JH Stroebel P Allgayer H. PMA Up-Regulates the Transcription of Axl by AP-1 Transcription Factor Binding to TRE Sequences *via* the MAPK Cascade in Leukaemia Cells. Biol Cell (2011) 103:21–33. doi: 10.1042/BC20100094 20977427

[92] Liu Y-N Chang T-H Tsai M-F Wu S-G Tsai T-H Chen H-Y. IL-8 Confers Resistance to EGFR Inhibitors by Inducing Stem Cell Properties in Lung Cancer. Oncotarget (2015) 6:10415–31. doi: 10.18632/oncotarget.3389 PMC4496364 25871388

[93] Karachaliou N Chaib I Cardona AF Berenguer J Bracht JWP Yang J. Common Co-Activation of AXL and CDCP1 in EGFR-Mutation-Positive Non-Small Cell Lung Cancer Associated With Poor Prognosis. EBioMedicine (2018) 29:112–27. doi: 10.1016/j.ebiom.2018.02.001 PMC5925453 29433983

[94] Peled N Wynes MW Ikeda N Ohira T Yoshida K Qian J. Insulin-Like Growth Factor-1 Receptor (IGF-1R) as a Biomarker for Resistance to the Tyrosine Kinase Inhibitor Gefitinib in Non-Small Cell Lung Cancer. Cell Oncol (Dordr) (2013) 36:277–88. doi: 10.1007/s13402-013-0133-9 PMC4186686 23619944

[95] Yeo CD Park KH Park CK Lee SH Kim SJ Yoon HK. Expression of Insulin-Like Growth Factor 1 Receptor (IGF-1R) Predicts Poor Responses to Epidermal Growth Factor Receptor (EGFR) Tyrosine Kinase Inhibitors in non-Small Cell Lung Cancer Patients Harboring Activating EGFR Mutations. Lung Cancer (2015) 87:311–7. doi: 10.1016/j.lungcan.2015.01.004 25617986

[96] Cortot AB Repellin CE Shimamura T Capelletti M Zejnullahu K Ercan D. Resistance to Irreversible EGF Receptor Tyrosine Kinase Inhibitors Through a Multistep Mechanism Involving the IGF1R Pathway. Cancer Res (2013) 73:834–43. doi: 10.1158/0008-5472.CAN-12-2066 PMC3994895 23172312

[97] Aghdassi A Sendler M Guenther A Mayerle J Behn C-O Heidecke C-D. Recruitment of Histone Deacetylases HDAC1 and HDAC2 by the Transcriptional Repressor ZEB1 Downregulates E-Cadherin Expression in Pancreatic Cancer. Gut (2012) 61:439–48. doi: 10.1136/gutjnl-2011-300060 22147512

[98] Brabletz S Brabletz T. The ZEB/miR-200 Feedback Loop—A Motor of Cellular Plasticity in Development and Cancer? EMBO Rep (2010) 11:670–7. doi: 10.1038/embor.2010.117 PMC2933868 20706219

[99] Vijay GV Zhao N Den Hollander P Toneff MJ Joseph R Pietila M. Gsk3β Regulates Epithelial-Mesenchymal Transition and Cancer Stem Cell Properties in Triple-Negative Breast Cancer. Breast Cancer Res (2019) 21:37. doi: 10.1186/s13058-019-1125-0 30845991 PMC6407242

[100] Feldker N Ferrazzi F Schuhwerk H Widholz SA Guenther K Frisch I. Genome-Wide Cooperation of EMT Transcription Factor ZEB 1 With YAP and AP -1 in Breast Cancer. EMBO J (2020) 39(17):e103209. doi: 10.15252/embj.2019103209 32692442 PMC7459422

[101] DeNardo DG Barreto JB Andreu P Vasquez L Tawfik D Kolhatkar N. CD4(+) T Cells Regulate Pulmonary Metastasis of Mammary Carcinomas by Enhancing Protumor Properties of Macrophages. Cancer Cell (2009) 16:91–102. doi: 10.1016/j.ccr.2009.06.018 19647220 PMC2778576

[102] Kim TM Song A Kim D-W Kim S Ahn Y-O Keam B. Mechanisms of Acquired Resistance to AZD9291. J Thorac Oncol (2015) 10:1736–44. doi: 10.1097/JTO.0000000000000688 26473643

[103] Arasada RR Amann JM Rahman MA Huppert SS Carbone DP. EGFR Blockade Enriches for Lung Cancer Stem–like Cells Through Notch3-Dependent Signaling. Cancer Res (2014) 74:5572–84. doi: 10.1158/0008-5472.CAN-13-3724 PMC4263272 25125655

[104] Codony-Servat C Codony-Servat J Karachaliou N Molina MA Chaib I Ramirez JL. Activation of Signal Transducer and Activator of Transcription 3 (STAT3) Signaling in EGFR Mutant non-Small-Cell Lung Cancer (NSCLC). Oncotarget (2017) 8:47305–16. doi: 10.18632/oncotarget.17625 PMC5564566 28521301

[105] Goto N Ueo T Fukuda A Kawada K Sakai Y Miyoshi H. Distinct Roles of HES1 in Normal Stem Cells and Tumor Stem-Like Cells of the Intestine. Cancer Res (2017) 77:3442–54. doi: 10.1158/0008-5472.CAN-16-3192 28536281

[106] Bousquet Mur E Bernardo S Papon L Mancini M Fabbrizio E Goussard M. Notch Inhibition Overcomes Resistance to Tyrosine Kinase Inhibitors in EGFR-Driven Lung Adenocarcinoma. J Clin Invest (2019) 130:612–24. doi: 10.1172/JCI126896 PMC6994195 31671073

[107] Faber AC Corcoran RB Ebi H Sequist LV Waltman BA Chung E. BIM Expression in Treatment-Naïve Cancers Predicts Responsiveness to Kinase Inhibitors. Cancer Discov (2011) 1:352–65. doi: 10.1158/2159-8290.CD-11-0106 PMC3229203 22145099

[108] Ley R Balmanno K Hadfield K Weston C Cook SJ. Activation of the ERK1/2 Signaling Pathway Promotes Phosphorylation and Proteasome-Dependent Degradation of the BH3-Only Protein, Bim. J Biol Chem (2003) 278:18811–6. doi: 10.1074/jbc.M301010200 12646560

[109] Domina AM Vrana JA Gregory MA Hann SR Craig RW. MCL1 is Phosphorylated in the PEST Region and Stabilized Upon ERK Activation in Viable Cells, and at Additional Sites With Cytotoxic Okadaic Acid or Taxol. Oncogene (2004) 23:5301–15. doi: 10.1038/sj.onc.1207692 15241487

[110] Bertino EM Gentzler RD Clifford S Kolesar J Muzikansky A Haura EB. Phase IB Study of Osimertinib in Combination With Navitoclax in *EGFR* -Mutant NSCLC Following Resistance to Initial *EGFR* Therapy (ETCTN 9903). Clin Cancer Res (2021) 27:1604–11. doi: 10.1158/1078-0432.CCR-20-4084 PMC7976451 33376097

[111] Shi P Zhang S Zhu L Qian G Ren H Ramalingam SS. The Third-Generation EGFR Inhibitor, Osimertinib, Promotes C-FLIP Degradation, Enhancing Apoptosis Including TRAIL-Induced Apoptosis in NSCLC Cells With Activating EGFR Mutations. Trans Oncol (2019) 12:705–13. doi: 10.1016/j.tranon.2019.02.006 PMC6411612 30856555

[112] Bivona TG Hieronymus H Parker J Chang K Taron M Rosell R. FAS and NF-κb Signalling Modulate Dependence of Lung Cancers on Mutant EGFR. Nature (2011) 471:523–6. doi: 10.1038/nature09870 PMC3541675 21430781

[113] Moiseeva O Deschênes-Simard X St-Germain E Igelmann S Huot G Cadar AE. Metformin Inhibits the Senescence-Associated Secretory Phenotype by Interfering With IKK / NF -κ B Activation. Aging Cell (2013) 12:489–98. doi: 10.1111/acel.12075 23521863

[114] Tang A Gao K Chu L Zhang R Yang J Zheng J. Aurora Kinases: Novel Therapy Targets in Cancers. Oncotarget (2017) 8:23937–54. doi: 10.18632/oncotarget.14893 PMC5410356 28147341

[115] Shi R Radulovich N Ng C Liu N Notsuda H Cabanero M. Organoid Cultures as Preclinical Models of Non-Small Cell Lung Cancer. Clin Cancer Res (2020) 26:1162–74. doi: 10.1158/1078-0432.CCR-19-1376 31694835

